# Protective Behaviors and Secondary Harms Resulting From Nonpharmaceutical Interventions During the COVID-19 Epidemic in South Africa: Multisite, Prospective Longitudinal Study

**DOI:** 10.2196/26073

**Published:** 2021-05-13

**Authors:** Guy Harling, Francesc Xavier Gómez-Olivé, Joseph Tlouyamma, Tinofa Mutevedzi, Chodziwadziwa Whiteson Kabudula, Ruth Mahlako, Urisha Singh, Daniel Ohene-Kwofie, Rose Buckland, Pedzisai Ndagurwa, Dickman Gareta, Resign Gunda, Thobeka Mngomezulu, Siyabonga Nxumalo, Emily B Wong, Kathleen Kahn, Mark J Siedner, Eric Maimela, Stephen Tollman, Mark Collinson, Kobus Herbst

**Affiliations:** 1 Africa Health Research Institute KwaZulu-Natal South Africa; 2 Institute for Global Health University College London London United Kingdom; 3 Medical Research Council/Wits Rural Public Health and Health Transitions Research Unit (Agincourt) School of Public Health, Faculty of Health Sciences University of the Witwatersrand Johannesburg South Africa; 4 Department of Epidemiology Harvard T.H. Chan School of Public Health Harvard University Boston, MA United States; 5 Center for Population and Development Studies Harvard T.H. Chan School of Public Health Harvard University Boston, MA United States; 6 School of Nursing and Public Health University of KwaZulu-Natal Durban South Africa; 7 International Network for the Demographic Evaluation of Populations and Their Health Network Accra Ghana; 8 Dikgale-Mamabolo-Mothiba Population Health Research Centre School of Health Care Sciences, Faculty of Health Sciences University of Limpopo Mankweng South Africa; 9 Department of Computer Science School of Mathematical and Computer Sciences, Faculty of Science and Agriculture University of Limpopo Mankweng South Africa; 10 Department of Science and Innovation-Medical Research Council South African Population Research Infrastructure Network Johannesburg South Africa; 11 Division of Infectious Diseases University of Alabama, Birmingham Birmingham, AL United States; 12 Harvard Medical School and the Medical Practice Evaluation Center Massachusetts General Hospital Boston, MA United States; 13 Department of Public Health School of Health Care Sciences, Faculty of Health Sciences University of Limpopo Mankweng South Africa

**Keywords:** behaviour change, COVID-19, economic well-being, health care access, health knowledge, mental health, South Africa, surveillance, nonpharmaceutical interventions

## Abstract

**Background:**

In March 2020, South Africa implemented strict nonpharmaceutical interventions (NPIs) to contain the spread of COVID-19. Over the subsequent 5 months, NPI policies were eased in stages according to a national strategy. COVID-19 spread throughout the country heterogeneously; the disease reached rural areas by July and case numbers peaked from July to August. A second COVID-19 wave began in late 2020. Data on the impact of NPI policies on social and economic well-being and access to health care are limited.

**Objective:**

We aimed to determine how rural residents in three South African provinces changed their behaviors during the first COVID-19 epidemic wave.

**Methods:**

The South African Population Research Infrastructure Network nodes in the Mpumalanga (Agincourt), KwaZulu-Natal, (Africa Health Research Institute) and Limpopo (Dikgale-Mamabolo-Mothiba) provinces conducted up to 14 rounds of longitudinal telephone surveys among randomly sampled households from rural and periurban surveillance populations every 2-3 weeks. Interviews included questions on the following topics: COVID-19–related knowledge and behaviors, the health and economic impacts of NPIs, and mental health. We analyzed how responses varied based on NPI stringency and household sociodemographics.

**Results:**

In total, 5120 households completed 23,095 interviews between April and December 2020. Respondents’ self-reported satisfaction with their COVID-19–related knowledge and face mask use rapidly rose to 85% and 95%, respectively, by August. As selected NPIs were eased, the amount of travel increased, economic losses were reduced, and the prevalence of anxiety and depression symptoms fell. When the number of COVID-19 cases spiked at one node in July, the amount of travel dropped rapidly and the rate of missed daily medications doubled. Households where more adults received government-funded old-age pensions reported concerns about economic matters and medication access less often.

**Conclusions:**

South Africans complied with stringent, COVID-19–related NPIs despite the threat of substantial social, economic, and health repercussions. Government-supported social welfare programs appeared to buffer interruptions in income and health care access during local outbreaks. Epidemic control policies must be balanced against the broader well-being of people in resource-limited settings and designed with parallel support systems when such policies threaten peoples’ income and access to basic services.

## Introduction

Since the emergence of COVID-19 in humans in late 2019, the epidemic has spread to every country in the world, resulting in direct mortality and morbidity [1] and indirect impacts on physical and mental health and economic well-being [2-4]. Shortly after COVID-19 was declared a Public Health Emergency of International Concern on January 30, 2020, South Africa was identified as a highly vulnerable country due to (1) its extensive internal and international transportation links [5]; (2) its burden of infectious and noncommunicable health conditions [6,7]; and (3) its large, socioeconomically vulnerable population [8]. The national government rapidly announced strict, nationwide nonpharmaceutical interventions (NPIs; Level 5 lockdown) on March 26, 2020. Under these NPIs, leaving home was only allowed for grocery shopping, obtaining medicine and medical care, or conducting permitted essential work. Furthermore, tobacco and alcohol sales were banned, and from May 1 onward, face mask use was mandatory in public spaces. These regulations were accompanied with guidance on enhanced handwashing, sanitizer use, and surface cleaning.

The lockdown was intended to (1) reduce COVID-19 transmission through strictly restricting physical interactions; (2) prevent rapid epidemic growth and allow health care providers to prepare for a subsequent rise in the demand for care; (3) promote widespread educational campaigns to reduce COVID-19 transmission; and (4) initiate an ambitious, country-wide, community-based COVID-19 screening and testing program [9]. Between May and September 2020, the lockdown was gradually eased, allowing people to return work and school and engage in limited public gatherings. For example, under Level 4 lockdown, restrictions were eased to allow the restarting of work in high-value sectors, meal deliveries, and nongroup daytime exercise. Under Level 3 lockdown, limited religious gatherings, professional noncontact sports, and sit-down meals were allowed.

Although the cumulative number of COVID-19 cases slowly grew during April 2020, the disease incidence curve rapidly rose from May onward, peaking between June and August. By September 1, 2020, South Africa had reported over 625,000 confirmed COVID-19 cases and over 14,000 deaths [10]; the COVID-19 epidemic in South Africa was among the 10 largest epidemics reported worldwide by that date [11]. The true impact of the epidemic appears to be even greater, with an excess of 42,396 deaths reported in South Africa between January and August 2020 compared to those reported in the same period in 2018 and 2019 [12].

The relaxation of lockdown regulations, which occurred even as the epidemic grew, reflected competing health and economic vulnerabilities and priorities as well as sustained popular pressures [13-15]. There was widespread concern that the lockdown was substantially affecting the national economy; individual household livelihoods; and access to education, health care, and medication [8,16]. Additionally, some expected the lockdown to be futile, since much of the population could neither maintain physical distancing nor implement NPIs due to household and community overcrowding and limited access to running water and sanitation [17].

Robust data are essential for evaluating the hypotheses that lockdowns cause substantial harm or are futile and for targeting locations that are the most in need of resources. The impact of NPIs has been evaluated in various low- and middle-income countries, generating evidence of early reductions in income and food security and the rapid, substantial uptake of protective behaviors [18-22].

Although South Africa has effective national health care surveillance systems, there is limited capacity for monitoring the social and behavioral effects of NPIs on the COVID-19 epidemic at a local level. NPIs, such as those implemented in South Africa, might be expected to generate differing risks and benefits across socioeconomic settings. For example, there was unease in rural areas regarding water access for hand hygiene and *imilindo* (funeral night vigils held in crowded rooms) [23], while people from urban areas worried about dwelling proximity and shared ablutions [24]. The number of robust comparisons between urban and rural settings has been limited, but such comparisons are vital if public sector responses are to be effectively aligned with prevailing conditions.

To date, most evaluations of the impact of COVID-19 in South Africa have been limited to web-based or urban settings [25-27]. Studies on the initial weeks of lockdown reported that although people had substantial concerns about the disease and its economic impact, they also had a strong willingness to abide by travel restrictions and other measures [26,27]. To date, the most comprehensive longitudinal study of the impact of the COVID-19 epidemic in South Africa on behavior is the National Income Dynamics Study (NIDS)-Coronavirus Rapid Mobile Survey (CRAM). The NIDS is an ongoing panel survey that began in 2008 and follows a nationally representative sample of households and their members [28]. The NIDS-CRAM recontacted NIDS respondents from the most recent interview wave in 2017 [29]. In 2020, 3 rounds of telephonic data collection were completed (first round: May and June; second round: July and August; third round: November and December), and data on a wide range of social and economic impacts of the epidemic were captured [25,30,31]. Employment dropped sharply after lockdown was imposed, but by October 2020 the overall employment rate appeared to have recovered, although not for women or respondents with low levels of education. The provision of increased government support, including top-ups for existing unconditional grants (which ended after October) and the new, temporary Social Relief of Distress grant for working-aged adults with no other sources of support appeared to aid households, particularly those in rural areas [32]. However, the withdrawal of these grants has caused concern. Mental health concerns remained substantial throughout the year and were positively associated with household child hunger [33].

The NIDS-CRAM has substantial strengths in terms of its national reach and wide-ranging topic coverage. However, it only provides sporadic snapshots of the epidemic. We therefore used an existing research infrastructure in three South African provinces to evaluate how health, social, and economic behaviors continuously changed between April and December 2020. We used a high-frequency survey of a panel of households for which substantial, pre-epidemic data were already available. Our first hypothesis was that behaviors would change as regulations and the national epidemic changed. Our second hypothesis was that these changes would vary based on socioeconomic characteristics (those that reflected households’ ability to maintain their compliance with NPI and social distancing policies) and households’ needs and resources.

## Methods

### Study Site

The South African Population Research Infrastructure Network (SAPRIN)—an initiative that is hosted by the South African Medical Research Council and receives long-term from the National Department of Science and Innovation—integrates three Health and Demographic Surveillance System (HDSS) nodes for population and health surveillance: (1) the Medical Research Council (MRC)/Wits Rural Public Health and Health Transitions Research Unit (Agincourt) in Ehlanzeni district, Mpumalanga [34]; (2) the Dikgale-Mamabolo-Mothiba (DIMAMO) Population Health Research Centre in Capricorn district, Limpopo [35]; and (3) the Africa Health Research Institute (AHRI) in uMkhanyakude district, KwaZulu-Natal [36]. Other urban nodes are under development. The nodes, which each contain over 100,000 individuals residing in approximately 20,000 households, vary in settlement structure and density. The three districts are rural or periurban areas that are located on the east side of South Africa (Figure S1 in Multimedia Appendix 1) and have low average income levels relative to the rest of the country. Nodes conduct multiple in-person and telephonic surveys per year to update health and sociodemographic data. However, DIMAMO had only partially captured socioeconomic data for the first time before the COVID-19 epidemic began in South Africa.

### Study Design and Implementation

In March 2020, SAPRIN initiated plans for each HDSS node to implement a high-intensity, longitudinal telephonic survey that covered at least 750 randomly selected households in each province, by using telephone numbers extracted from each node’s most recent census. This sample was selected to obtain estimates of survey- and wave-specific proportions with a precision of no less than 4 percentage points, under the assumption of an 80% response rate based on past SAPRIN surveys. Every 2-3 weeks, a central call center at each node contacted households and asked a primary respondent to answer questions on behalf of the household. Primary respondents had to be resident adult members of the household (aged ≥18 years). To combat survey fatigue, from mid-September onward or after the seventh survey wave (whichever was earlier), one-third of the cohort was rotated out at each subsequent wave, and a new random sample that included the same number of households was rotated in for 4-6 survey waves. Details for each node are shown in Figure S2 in Multimedia Appendix 1.

The questionnaire included both household-level and individual-specific questions; the latter could be directly answered by other household members if they were present. Otherwise, the primary respondent served as a proxy. The questionnaire included COVID-19 symptom screening; individuals who met the Department of Health’s COVID-19 symptom criteria were referred for further investigation, possible testing, and care. Data were captured on laptop computers by call center interviewers who used electronic data capture software, including automated skip patterns and validation checks. Telephone calls continued from April to December 2020 with continuous quality monitoring. The survey implementation process at one node (AHRI) is described in detail elsewhere [37]. The study questionnaire is provided in Multimedia Appendix 1.

### Outcomes

Our outcomes for this study were based on answers to questions related to COVID-19 and NPIs in three key domains: (1) COVID-19–related knowledge and behavior; (2) the health and economic impacts of NPIs; and (3) mental health. For behavior, the primary respondent was asked to rate their perceived knowledge about COVID-19 on a 5-point scale; we classified respondents as (1) those who self-reported that they did not have enough knowledge or (2) those who reported that they had enough or more than enough knowledge. Respondents were then asked about household behavior changes that they made in response to the COVID-19 epidemic. They were asked whether any resident household member had left the house in the past 7 days and whether any nonhousehold members had visited the house during the preceding day; we classified respondents as either (1) those who reported any number of household or nonhousehold members or (2) those who reported “none” for each question. Respondents were also asked if household members had, over the past 7 days, avoided crowded areas or social events, travelled (using local minibus taxis or long-distance travel methods), or used face masks when going out.

For health and economic impacts, primary respondents were asked about household members’ ability to (1) access all needed daily medications, (2) access needed health care, and (3) earn money. Finally, for mental health, we asked primary respondents to answer the Generalized Anxiety Disorder 2-item (GAD-2) and Patient Health Questionnaire 2-item (PHQ-2) scales. GAD-2 and PHQ-2 scores of ≥3 were considered positive, as per the standard, South African–validated cutoffs [38]. The Cronbach α values in this study were .85 for the PHQ-2 and .91 for the GAD-2.

### Statistical Analysis

We linked data from the high-intensity SAPRIN survey to the following routine individual and household sociodemographic data, which were collected from households in 2019: the number of children, working-aged adults, and pension-aged adults; the maximum education level attained; node-specific asset index quintiles; levels of employment; and the receipt of unconditional social grants. South African noncontributory pensions are available to all citizens, permanent residents, or documented refugees aged >60 years, although pension applications require proof of status.

In this study, we included anyone who was interviewed in 2020. First, we described survey response rates at each node and time period as well as key, pre–COVID-19 epidemic household characteristics. Second, we described changes in each of the 11 measures across the three domains (behavior, health and economic impact, and mental health) based on node and month of data collection. Third, we used multivariable regression models to assess independent predictors of our outcomes of interest via complete case analysis. For each outcome, we fitted a Poisson model with household-level random effects and robust SEs to calculate prevalence ratios. All models included variables for node, interview round number, month of the interview, and household characteristics. Data analysis was conducted with Stata version 15.1 (StataCorp LLC) and R version 4.0.2 (The R Foundation) [39]. Results were considered statistically significant at the .05 level.

### Ethical Considerations

All households previously provided consent to be contacted by phone and each primary respondent provided recorded, verbal consent. Households were not directly compensated for study participation; however, one node annually provided a shopping voucher (value of around US $3) to each participating household to thank them for their participation in all SAPRIN-related activities. Responses to questions were electronically captured in secure, on-premise databases with role-based security. Personally identifiable data were sequestered in separate database tables with restricted access, and all analytic data sets were pseudonymized prior to analysis. All study procedures were approved by Limpopo, Mpumalanga, and KwaZulu-Natal’s provincial Department of Health Research Ethics Committees (RECs), the University of KwaZulu-Natal’s Biomedical REC, the University of the Witwatersrand’s Human REC (Medical), and the University of Limpopo’s Turfloop REC.

### Patient and Public Involvement

The adaptation of the existing SAPRIN surveillance program was discussed with and approved by each nodes’ existing community advisory groups prior to the finalization of the study protocol. The results of the studies were routinely shared with the community through a range of engagement activities that were conducted by the teams at each node.

### Data Availability

The data collected in the SAPRIN COVID-19 surveillance survey, including those reported in this paper, will be made available in pseudonymized form through the SAPRIN data repository [40].

## Results

### Study Implementation

**Figure 1:**
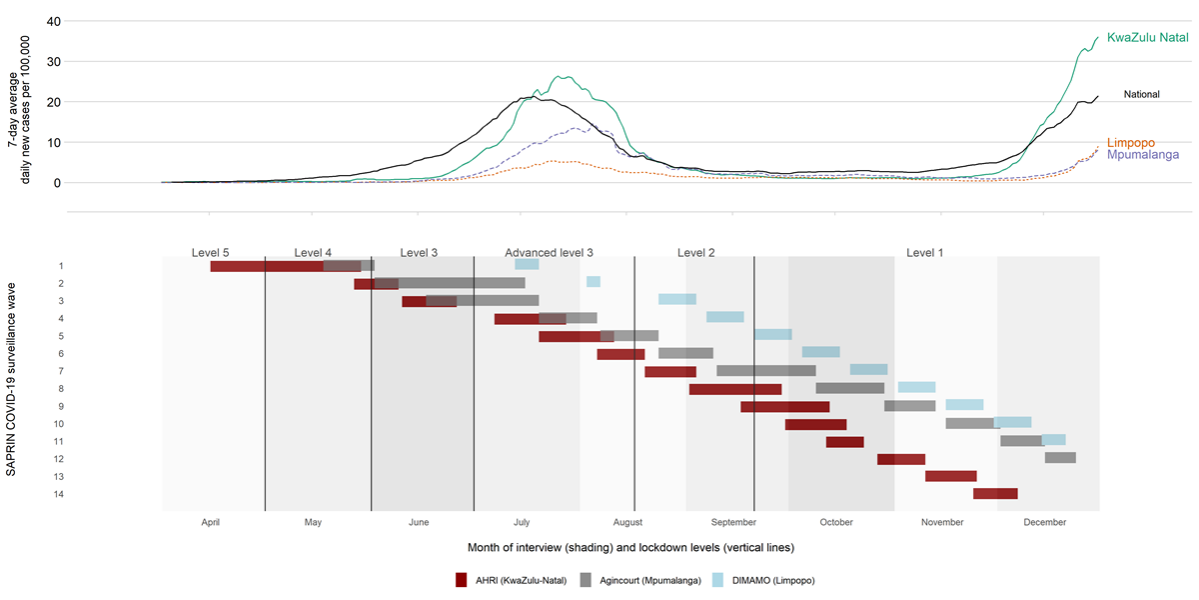
Epidemic curve and interview rounds across SAPRIN nodes in 2020. AHRI: Africa Health Research Institute; DIMAMO: Dikgale-Mamabolo-Mothiba; SAPRIN: South African Population Research Infrastructure Network.

Between April 15 and December 24, 2020, AHRI (in KwaZulu-Natal) completed 14 waves of data collection, Agincourt (in Mpumalanga) completed 12 waves of data collection, and DIMAMO (in Limpopo) completed 11 waves of data collection (Figure 1). These waves covered the entire period of the first COVID-19 epidemic wave in South Africa, including outbreaks of varying sizes that occurred in the three provinces under observation, and part of the early phase of the second national wave. The average response rate was 71% (23,095/31,643), and response rates varied from 56.9% (427/750) to 90.3% (1013/1122) depending on wave and node. Direct refusal was rare (1242/31,643, 3.8%), while unanswered calls were more common (4304/31,643, 13.2%). Phone numbers were quite often out of service or claimed to be wrong numbers (3002/31,643, 9.2%; Table S1 in Multimedia Appendix 1). Nonresponding households had lower levels of maximum education and fewer employed members, but responding and nonresponding households did not differ greatly based on household wealth or grant receipt (based on 2019 data; Table S2 in Multimedia Appendix 1). In total, 23,095 household interviews were completed with 5120 unique households in 2020.

### Descriptive Results

Descriptive statistics for all respondent households with valid telephone numbers and members who consented to and completed an interview are shown in Table 1. In one-sixth (894/3932, 21.7%) of the households, no one had completed secondary education, while 19.3% (797/3932) included a household member who had completed a postsecondary qualification. Wealthier households were more likely to have a valid telephone number; at the AHRI and Agincourt nodes they were also more likley to participate in study surveys. Households were large (median of 5 resident and nonresident members, of whom 2 were aged <18 years). Households had a mean of 1.2 employed members. In total, 38.9% (1979/5083) of all households received 1 or more old-age pensions and 66.2% (3364/5083) received other government grants.

**Table 1:** Descriptive statistics for participating households at South African Population Research Infrastructure Network nodes from April to December 2020.

Characteristics			Total^a^		Node^b^							
					AHRI^c^		Agincourt		DIMAMO^d^		*P* value^e^	
Number of households			5120		1608		1797		1715		N/A^f^	
**Highest education attained, %^g^**												<.001
	Less than complete secondary education	21.7		23.4		22.5		16.3				
	Complete secondary education	59		59.7		77.3		17.3				
	Diploma/certificate/degree	19.3		16.9		0.2		66.4				
**Node-specific household wealth quintile, %^h^**												<.001
	Lowest	14		16.3		9.6		18.1				
	Second lowest	18.2		19.8		16.5		18.4				
	Middle	20.9		20.1		20.8		22.6				
	Second highest	22.8		20.4		25.4		22.2				
	Highest	24.1		23.5		27.7		18.6				
**Household size in 2020^i^, median (IQR)**			5 (3-8)		5 (3-8)		6 (4-8)		5 (3-7)		<.001	
	Number of children	2 (1-3)		2 (1-4)		2 (1-3)		1 (0-3)		<.001		
	Number of working age adults	3 (2-4)		2 (1-4)		3 (2-5)		3 (2-4)		<.001		
	Number of people aged over 60	0 (0-1)		0 (0-1)		0 (0-1)		0 (0-1)		.79		
Number of full-time or part-time employed people, median (IQR)^j^			1 (0-2)		1 (0-2)		1 (0-2)		0 (0-1)		<.001	
Number of pension grant receivers, median (IQR)^j^			0 (0-1)		0 (0-1)		0 (0-1)		0 (0-1)		.06	
Number of nonpension grant receivers, median (IQR)^j^			1 (0-3)		1 (0-3)		2 (0-3)		1 (0-2)		<.001	

^a^Percentages refer to all participating households.

^b^Percentages refer to all households with nonmissing values.

^c^AHRI: Africa Health Research Institute.

^d^DIMAMO: Dikgale-Mamabolo-Mothiba

^e^*P* values were based on the differences in characteristics between groups and were calculated from Chi-square tests for categorical variables and Wilcoxon rank-sum tests (participation comparison) or Kruskal-Wallis tests (node comparisons) for continuous variables.

^f^N/A: not applicable.

^g^Data are missing from 1.4% (22/1608) of households surveyed by AHRI, 4.8% (86/1797) of households surveyed by Agincourt, and 48.3% (828/1715) of households surveyed by DIMAMO.

^h^Data are missing from 0.5% (8/1608) of households surveyed by AHRI, 6.6% (118/1797) households surveyed by Agincourt, and 47.7% (818/1715) of households surveyed by DIMAMO.

^i^Household sizes in 2020 were only determined for participating households.

^j^Data are missing from 23 households surveyed by AHRI, 10 households surveyed by Agincourt, and 4 households surveyed by DIMAMO.

**Figure 2:**
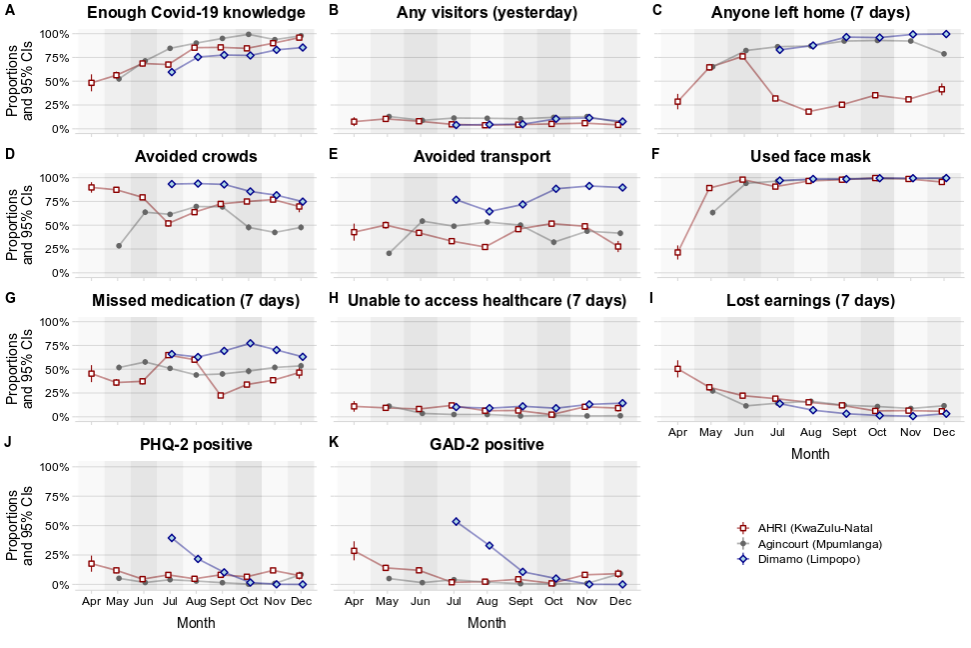
Knowledge about, behavior relating to, and impact of COVID-19 and related regulations at South African Population Research Infrastructure Network nodes from April to December 2020. Graphs A, D, E, F, J, and K reflect individual-level responses of the primary household respondents. Graphs B, C, G, H, and I reflect household-level responses reported by the primary household respondents. Values are proportions of household primary respondents and 95% CIs. Precise values are provided in Table S3 in Multimedia Appendix 1. AHRI: Africa Health Research Institute; DIMAMO: Dikgale-Mamabolo-Mothiba; GAD-2: Generalized Anxiety Disorder 2-item; PHQ-2: Patient Health Questionnaire 2-item.

Figure 2 describes the questionnaire responses across time and location. Respondents’ self-reported satisfaction with their knowledge about COVID-19 rose over time at all three nodes from 53.7% (972/1811) in April and May 2020 to 92.1% (1997/2169) in December 2020. The number of daily visitors to households was consistently low; the proportion of households that had 1 or more visitors on the day before the survey peaked in May (202/1693, 11.9%). The proportion of households with members who left home increased over time, ranging from 28.2% (33/117) in April to a peak of 79.5% (1459/1836) in June. There was however a notable drop in this proportion at AHRI in KwaZulu-Natal (June: 641/842, 76.1%; August: 192/1064, 18%) that occurred concurrently with reports of local COVID-19 transmission. The proportion somewhat increased again by October (400/1133, 35.3%). Face mask use rose rapidly and became almost universal at all three nodes by June.

Respondents’ reported inability to access health care remained low and relatively stable over time. However, households reported that members missed daily medications in the past week at 52.9% (12,155/22,974) of interviews. Although rates of missed daily medications were stable over time at Agincourt and DIMAMO, they almost doubled at AHRI from 37.4% (315/843) in June to 64.9% (634/979) in July, as the epidemic arrived in the local area. The proportion of households that reported that members lost earnings due to COVID-19 regulations dropped substantially as the lockdown was reduced from Level 5 to Level 4. This proportion dropped again when the lockdown was lowered to Level 3 and remained steadily low thereafter. Finally, the proportion of individuals who screened positive for possible anxiety and depression fell over time at AHRI and DIMAMO and stayed low at Agincourt throughout the period studied.

### Multivariable Results

After accounting for study node and month of interview, and despite variation in household composition, no household characteristics were substantively associated with (1) respondents self-reporting satisfaction with their COVID-19–related knowledge; (2) residents leaving their homes in the week before the interview; or (3) residents avoiding travel or face mask use (Tables 2 and 3). Households with individuals who had attained more education were more likely to report that they had had visitors on the day before the interview and less likely to report that they had avoided crowded spaces. These associations may reflect that more educated individuals are likelier to live in more urban locations.

**Table 2:** COVID-19–related knowledge and home behaviors in South Africa from April to August 2020^a^.

Variable			Individual has enough knowledge (n=17,292), prevalence ratio (95% CI)		Household had any visitors (n=17,282), prevalence ratio (95% CI)		Any member left home (n=17,164), prevalence ratio (95% CI)
**Node**							
	AHRI^b^ (KwaZulu-Natal)	1.00 (N/A^c^)		1.00 (N/A)		1.00 (N/A)	
	Agincourt (Mpumalanga)	1.09 (1.07-1.11)		2.07 (1.76-2.43)		2.26 (2.18-2.35)	
	DIMAMO^d^ (Limpopo)	0.91 (0.88-0.95)		1.42 (1.07-1.88)		2.58 (2.46-2.72)	
**Month of interview**							
	April	0.93 (0.77-1.13)		1.08 (0.57-2.04)		0.76 (0.57-1.02)	
	May	1.00 (N/A)		1.00 (N/A)		1.00 (N/A)	
	June	1.32 (1.25-1.39)		0.72 (0.59-0.88)		1.29 (1.24-1.35)	
	July	1.45 (1.38-1.52)		0.71 (0.60-0.84)		0.98 (0.94-1.03)	
	August	1.64 (1.56-1.71)		0.64 (0.53 0.77)		0.91 (0.87-0.96)	
	September	1.69 (1.62-1.77)		0.69 (0.57-0.83)		1.00 (0.95-1.04)	
	October	1.71 (1.63-1.79)		0.79 (0.65-0.96)		1.05 (1.00-1.10)	
	November	1.72 (1.64-1.80)		0.87 (0.72-1.05)		1.03 (0.98-1.08)	
	December	1.80 (1.71-1.88)		0.48 (0.37-0.62)		0.98 (0.93-1.03)	
**Household members (per person)**							
	Children	1.00 (1.00-1.01)		0.96 (0.91-1.01)		1.02 (1.02-1.03)	
	Working-aged adults	1.00 (1.00-1.00)		0.99 (0.96-1.03)		1.02 (1.01-1.02)	
	Pension-aged adults	0.98 (0.96-1.00)		1.09 (0.95-1.26)		1.01 (0.98-1.04)	
**Maximum education of household members**							
	Less than complete secondary education	1.00 (N/A)		1.00 (N/A)		1.00 (N/A)	
	Complete secondary education	1.02 (1.00-1.04)		0.92 (0.78-1.08)		1.00 (0.97-1.03)	
	Diploma/certificate/degree	1.02 (0.98-1.06)		1.06 (0.80-1.39)		1.01 (0.96-1.07)	
**Household income sources in 2019**							
	Full-time and part-time employees	1.01 (1.00-1.01)		1.06 (1.01-1.11)		1.01 (1.00-1.02)	
	Pension grants	0.99 (0.97-1.01)		0.96 (0.83 - 1.11)		0.97 (0.94 - 1.00)	
	Nonpension grants	1.00 (1.00-1.01)		0.99 (0.94-1.04)		0.99 (0.99-1.00)	
Household asset index quintiles			1.00 (0.99-1.01)		0.99 (0.94-1.03)		1.00 (1.00-1.01)

^a^Each column (knowledge, the number of visitors, and members leaving home) is a single Poisson regression with household-level random effects and robust SEs for calculating prevalence ratios and 95% CIs. In total, 17,384 observations have complete covariate data and 5704 observations from 1475 households have missing covariate values; 33 households surveyed by AHRI, 103 surveyed by Agincourt, and 1339 surveyed by DIMAMO are missing data. The remaining missing observations reflect missing outcome values.

^b^AHRI: Africa Health Research Institute.

^c^N/A: not applicable.

^d^DIMAMO: Dikgale-Mamabolo-Mothiba

**Table 3:** Beyond-home behavior regarding COVID-19 at South African Population Research Infrastructure Network nodes from April to August 2020^a^.

Variable		Individual avoided crowds (n=17,043), prevalence ratio (95% CI)	Individual avoided transport (n=17,043), prevalence ratio (95% CI)	Individual used face mask (n=17,043), prevalence ratio (95% CI)
**Node**				
	AHRI^b^ (KwaZulu-Natal)	1.00 (N/A^c^)	1.00 (N/A)	1.00 (N/A)
	Agincourt (Mpumalanga)	0.77 (0.75-0.79)	1.05 (1.01-1.10)	0.98 (0.97-0.99)
	DIMAMO^d^ (Limpopo)	1.24 (1.19-1.29)	1.87 (1.76-1.98)	1.00 (0.98-1.01)
**Month of interview**				
	April	1.51(1.39-1.63)	1.40 (1.12-1.75)	0.28 (0.20-0.40)
	May	1.00 (N/A)	1.00 (N/A)	1.00 (N/A)
	June	1.36 (1.29-1.43)	1.54 (1.41-1.68)	1.30 (1.26-1.34)
	July	1.15 (1.09-1.22)	1.37 (1.26-1.49)	1.28 (1.24-1.32)
	August	1.30 (1.23-1.38)	1.31 (1.20-1.43)	1.32 (1.28-1.36)
	September	1.35 (1.28-1.42)	1.48 (1.36-1.60)	1.33 (1.30-1.37)
	October	1.18 (1.12-1.25)	1.40 (1.29-1.52)	1.34 (1.31-1.38)
	November	1.13 (1.07-1.19)	1.48 (1.36-1.61)	1.34 (1.30-1.38)
	December	1.08 (1.01-1.15)	1.30 (1.19-1.43)	1.34 (1.30-1.39)
**Household members (per person)**				
	Children	1.00 (1.00-1.01)	1.01 (1.00-1.02)	1.00 (1.00-1.00)
	Working-aged adults	1.02 (1.01-1.02)	1.02 (1.01-1.03)	1.00 (1.00-1.00)
	Pension-aged adults	1.02 (1.00-1.05)	1.00 (0.96-1.04)	1.00 (0.99-1.01)
**Maximum education of household members**				
	Less than complete secondary education	1.00 (N/A)	1.00 (N/A)	1.00 (N/A)
	Complete secondary education	1.00 (0.97-1.03)	1.02 (0.98-1.07)	1.00 (0.99-1.01)
	Diploma/certificate/degree	0.96 (0.92-1.00)	1.00 (0.94-1.07)	1.00 (0.98-1.01)
**Household income sources in 2019**				
	Full-time and part-time employees	0.99 (0.98-1.00)	0.99 (0.98-1.00)	1.00 (1.00-1.00)
	Pension grants	1.00 (0.97-1.02)	1.00 (0.97-1.05)	1.00 (0.99-1.01)
	Nonpension grants	1.00 (0.99-1.00)	0.99 (0.98-1.00)	1.00 (1.00-1.00)
Household asset index quintiles		1.00 (0.99-1.01)	1.00 (0.99-1.01)	1.00 (1.00-1.00)

^a^Each column (knowledge, the number of visitors, and members leaving home) is a single Poisson regression with household-level random effects and robust SEs for calculating prevalence ratios and 95% CIs. In total, 17,384 observations have complete covariate data and 5704 observations from 1475 households have missing covariate values; 33 households surveyed by AHRI, 103 surveyed by Agincourt, and 1339 surveyed by DIMAMO are missing data. The remaining missing observations reflect missing outcome values.

^b^AHRI: Africa Health Research Institute.

^c^N/A: not applicable.

^d^DIMAMO: Dikgale-Mamabolo-Mothiba

Households with a higher number of older individuals and pension recipients were more likely to have a recent unmet health need (nonsignificantly for older members; *P*=.55) but were less likely to have been unable to access medicine (Table 4). These same two factors predicted a lower prevalence of lost earnings, as did having a household member who had completed secondary education; having a household member who had completed postsecondary education was not predictive of lost earnings. Finally, the prevalence of depression and anxiety symptoms was higher in households with a member that had a postsecondary qualification, and the prevalence of depression symptoms was nonsignificantly (*P*=.18) greater in households that received pension grants compared to those that did not receive pension grants (either due to households having no eligible members or not having applied for such grants; Table 5).

**Table 4:** Health care and economic behaviors at South African Population Research Infrastructure Network nodes from April to August 2020^a^.

Variable		Any member missed daily medication (n=17,277), prevalence ratio (95% CI)	Any member unable to access health care (n=17,272), prevalence ratio (95% CI)	Any member lost earnings (n=17,256), prevalence ratio (95% CI)
**Node**				
	AHRI^b^ (KwaZulu-Natal)	1.00 (N/A^c^)	1.00 (N/A)	1.00 (N/A)
	Agincourt (Mpumalanga)	1.20 (1.14-1.27)	0.34 (0.29-0.40)	0.90 (0.80-1.01)
	DIMAMO^d^ (Limpopo)	1.60 (1.48-1.74)	1.66 (1.31-2.09)	0.27 (0.19-0.38)
**Month of interview**				
	April	1.08 (0.88-1.33)	0.64 (0.38-1.09)	1.56 (1.29-1.89)
	May	1.00 (N/A)	1.00 (N/A)	1.00 (N/A)
	June	1.06 (0.99-1.13)	0.51 (0.41-0.64)	0.58 (0.52-0.66)
	July	1.22 (1.15-1.28)	0.52 (0.42-0.64)	0.57 (0.52-0.64)
	August	1.10 (1.04-1.17)	0.35 (0.28-0.44)	0.57 (0.51-0.64)
	September	0.81 (0.76-0.87)	0.33 (0.26-0.42)	0.42 (0.37-0.48)
	October	0.93 (0.87-1.00)	0.17 (0.13-0.23)	0.27 (0.23-0.33)
	November	0.98 (0.92-1.05)	0.48 (0.39-0.59)	0.27 (0.22-0.32)
	December	1.02 (0.94-1.10)	0.48 (0.37-0.63)	0.37 (0.31-0.45)
**Household members (per person)**				
	Children	0.99 (0.98-1.01)	1.00 (0.96-1.04)	1.01 (0.97-1.04)
	Working-aged adults	0.98 (0.96-0.99)	1.03 (0.99-1.07)	1.07 (1.04-1.10)
	Pension-aged adults	0.81 (0.76-0.86)	1.04 (0.91-1.20)	0.82 (0.72-0.92)
**Maximum education of household members**				
	Less than complete secondary education	1.00 (N/A)	1.00 (N/A)	1.00 (N/A)
	Complete secondary education	1.07 (1.00-1.14)	0.98 (0.82-1.16)	1.15 (1.00-1.32)
	Diploma/certificate/degree	1.08 (0.99-1.18)	1.02 (0.82-1.28)	1.03 (0.83 - 1.28)
**Household income sources in 2019**				
	Full-time and part-time employees	1.02 (1.00-1.04)	1.01 (0.96-1.05)	1.04 (1.00-1.08)
	Pension grants	0.92 (0.86-0.98)	1.21 (1.05-1.40)	0.92 (0.81-1.05)
	Nonpension grants	1.01 (0.99-1.02)	0.98 (0.94-1.02)	1.04 (1.00-1.07)
Household asset index quintiles		1.01 (0.99-1.03)	1.00 (0.95-1.05)	0.99 (0.95-1.03)

^a^Each column (knowledge, the number of visitors, and members leaving home) is a single Poisson regression with household-level random effects and robust SEs for calculating prevalence ratios and 95% CIs. In total, 17,384 observations have complete covariate data and 5704 observations from 1475 households have missing covariate values; 33 households surveyed by AHRI, 103 surveyed by Agincourt, and 1339 surveyed by DIMAMO are missing data. The remaining missing observations reflect missing outcome values.

^b^AHRI: Africa Health Research Institute.

^c^N/A: not applicable.

^d^DIMAMO: Dikgale-Mamabolo-Mothiba

**Table 5:** Mental health impacts of COVID-19 and household characteristics at South African Population Research Infrastructure Network nodes from April to August 2020^a^.

Variable			Individual PHQ-2^b^ screened positive (n=17,257), prevalence ratio (95% CI)		Individual GAD-2^c^ screened positive (n=17,256), prevalence ratio (95% CI)
**Node**					
	AHRI^d^ (KwaZulu-Natal)	1.00 (N/A^e^)		1.00 (N/A)	
	Agincourt (Mpumalanga)	0.35 (0.30-0.41)		0.46 (0.39-0.54)	
	DIMAMO^f^ (Limpopo)	1.16 (0.93-1.44)		2.83 (2.23-3.60)	
**Month of interview**					
	April	1.34 (0.87-2.05)		2.15 (1.55-2.99)	
	May	1.00 (N/A)		1.00 (N/A)	
	June	0.37 (0.27-0.50)		0.72 (0.57-0.91)	
	July	0.93 (0.76-1.14)		0.61 (0.50-0.75)	
	August	0.59 (0.47-0.74)		0.41 (0.33-0.51)	
	September	0.56 (0.44-0.71)		0.25 (0.19-0.33)	
	October	0.34 (0.26-0.46)		0.06 (0.04-0.10)	
	November	0.60 (0.47-0.77)		0.32 (0.25-0.43)	
	December	0.88 (0.67-1.16)		0.63 (0.47-0.84)	
**Household members (per person)**					
	Children	1.01 (0.97-1.05)		1.03 (0.98-1.07)	
	Working-aged adults	1.03 (0.99-1.07)		1.01 (0.97-1.05)	
	Pension-aged adults	0.88 (0.76-1.02)		0.91 (0.78-1.07)	
**Maximum education of household members**					
	Less than complete secondary	1.00 (N/A)		1.00 (N/A)	
	Complete secondary	1.07 (0.91-1.27)		0.89 (0.75-1.07)	
	Diploma/certificate/degree	1.33 (1.08-1.65)		1.16 (0.92-1.46)	
**Household income sources in 2019**					
	Full/part-time employees	0.97 (0.92-1.02)		0.98 (0.93-1.03)	
	Pension grants	1.11 (0.95-1.29)		0.99 (0.84-1.16)	
	Non-pension grants	0.98 (0.94-1.02)		0.99 (0.95-1.03)	
	**Household asset index quintiles**	1.01 (0.96-1.06)		1.06 (1.00-1.11)	

^a^Each column (knowledge, the number of visitors, and members leaving home) is on a single Poisson regression with household-level random effects and robust SEs for calculating prevalence ratios and 95% CIs. In total, 17,384 observations have complete covariate data and 5704 observations from 1475 households have missing covariate values; 33 households surveyed by AHRI, 103 surveyed by Agincourt, and 1339 households surveyed by DIMAMO are missing data. The remaining missing observations reflect missing outcome values.

^b^PHQ-2: Patient Health Questionnaire 2-item.

^c^GAD-2: Generalized Anxiety Disorder 2-item.

^d^AHRI: Africa Health Research Institute.

^e^N/A: not applicable.

^f^DIMAMO: Dikgale-Mamabolo-Mothiba

## Discussion

### Principal Findings

By conducting rapid, repeated telephonic interviews at sites in three provinces in South Africa, we observed how households in rural and periurban areas responded to and were affected by national NPIs that were enacted to minimize the epidemic spread of COVID-19. As both NPIs and the epidemic spread across the country, our longitudinal surveillance program captured the impact of both processes.

Our first key finding was that the South African national public health measures and messages implemented were effective in several ways. Respondents across three provinces showed consistent improvements over time in satisfaction with their understanding of the epidemic. There was early concern that South Africa’s public health messages were insufficiently contextualized to the country’s varying social and economic conditions. Both politicians and scientists conducted televised national press conferences that were supported by provincial and local follow-up events. Although causality cannot be proven between these events and changes in behavior, in line with other evidence from South Africa and other countries [21,31], our respondents reported that they rapidly and comprehensively complied with public health messages, including those about face mask use, and actively avoided crowds and public transport. Several of these protective behaviors remained prevalent even as formal, lockdown-related NPIs were relaxed and even after the first national epidemic wave had passed. This continued adherence to policies, which persisted even after they were no longer formally required, highlights the importance of considering how infection-related fears and prosocial desires to protect others can drive epidemic dynamics [41-43]; formal lockdowns may be less vital than carefully crafted public health communication.

Second, we identified substantial behavior changes as the COVID-19 epidemic arrived in the local study areas. This was particularly noticeable at the KwaZulu-Natal node, where rapid epidemic growth in the local district during early July coincided with a rapid decline in the proportion of household members leaving home and concomitant increases in levels of missed daily medications and the inability to access needed health care. There were also smaller behavior changes that occurred after mid-December at the Mpumalanga and Limpopo nodes (the KwaZulu-Natal node stopped data collection early in the month) as the second epidemic wave spread across the country.

These behavioral responses reflected local epidemic dynamics. The first national epidemic started in the Western Cape and spread first to the adjacent Eastern Cape before spreading to the densely populated Gauteng province and urban eThekwini in KwaZulu-Natal and then finally reaching the rural eastern and northern areas of South Africa, which were analyzed in this study. The second wave began in the Eastern Cape during November before spreading nationwide. The patterns of rapid behavior change in the face of a rising epidemic wave seen in this study were congruent with those seen worldwide. However, there are limited data on behavior in low- and middle-income countries either after first waves have receded or during subsequent waves. The impact of regulations and epidemic trajectories on travel is particularly pertinent in our study settings, as medium- and long-distance circular labor migration to urban areas is highly prevalent and vital to the economic well-being of rural and periurban South African households [44]. Bans on long-distance travel have potentially substantial economic implications for people who are not able to return to work, although such travel bans might also partially explain the limited epidemics that were seen in these rural areas even as NPIs were relaxed.

Third, our mental health findings are reassuring. At all three nodes, we observed declines over time in the prevalence of depression and anxiety symptoms (based on validated screening scales). The prevalence of such symptoms was notably higher at the DIMAMO periurban node in Limpopo earlier in the year. This higher prevalence perhaps reflected concerns of being at greater risk of SARS-CoV-2 infection due to respondents’ proximity to the nearby city of Polokwane. However, it was encouraging that even when the COVID-19 epidemic arrived at AHRI in KwaZulu-Natal during July 2020, depression and anxiety prevalence did not increase, though the uptick in these rates that occurred late in the year as the second wave arrived at Agincourt in Mpumalanga may be concerning. Mental health concerns were much more prevalent at some sites than at others, particularly earlier in the year, but it is also notable that households with postsecondary-educated members were significantly more likely to report depression (prevalence ratio: 1.33; 95% CI 1.08-1.65; *P*=.008) and nonsignificantly more likely to report anxiety (prevalence ratio: 1.16; 95% CI 0.92-1.46; *P*=.21). Comparisons are complex, but our findings align with national South African data, which suggest that COVID-19–related mental health impacts were more limited in low-income and rural areas [31]. Longitudinal surveillance across a range of settings via harmonized measures will help determine the extent to which mental health is directly affected by COVID-19–related fears and indirectly affected by secondary social and economic effects.

Fourth, our analysis raises concern about unmet needs for health care. Households reported that members had recently missed daily medication doses at almost half of all interviews (10,819/22,974, 47.1%) and that a member had wanted but was unable to access health care in the 7 days before the interview 6.7% (1538/22,967) of the time. These levels are similar to those of other South African surveys [25]. Notably, the epidemic’s arrival at AHRI had diverging effects; unmet health care needs did not change much, but missed medication rates almost doubled. These patterns suggest that household members may be calculating the trade-offs between COVID-19 and non–COVID-19 risks and are potentially willing to risk physical proximity to others to attend clinics [45] but not to collect medicine [46]. However, unmet health care and medication needs at the other study sites were stable throughout the observation period. Additional information is needed to determine (1) whether unmet health care needs are indicative of operational, mobility, transport cost, and transport availability issues or other issues; and (2) the extent to which such needs were the result of or were exacerbated by the COVID-19 epidemic or related regulations. Data that cover the pre-epidemic and postepidemic periods would help identify these effects, as would qualitative investigations of household decision making during lockdowns.

Finally, we found that households with higher numbers of older members and pension recipients reported more unmet health care needs but fewer instances of missed daily medication or lost earnings. South African noncontributory pensions—broad national government support schemes that are often the largest household income source in these relatively rural settings with very high unemployment rates—have previously been linked to positive physical and mental health outcomes [47,48]. Our study suggests that such government support structures likely play an essential role in maintaining household security in crisis contexts such as the COVID-19 epidemic by providing a guaranteed income to vulnerable populations. The government’s temporary supplementation of grant programs through top-up payments and novel noncontributory unemployment support early in the epidemic may have also helped [13]. However, it will be important to observe if the ending of income supplementation (grant top-ups ended in October 2020; unemployment payments have continued in 2021) reverses these supplements’ beneficial effects. The lack of substantive associations between household characteristics and social distancing measures is also noteworthy. In some instances, such as mask use, this likely reflects an overwhelming uptake of protective behaviors, which made statistically significant associations impossible. In other instances, our results suggest either that behavior was primarily driven by the epidemic cycle—as measured by interview month—or that key drivers of household behavior were not included in our analysis. Further investigations of this and other data sources may help determine which (if any) characteristics predict changes in protective behaviors during the COVID-19 epidemic.

This study presents an overview of key insights across time from multiple sites across South Africa. However, there are several additional analyses that could further contextualize our findings. First, data can be longitudinally analyzed at the household or individual level to evaluate trajectories of behavior and impact as the COVID-19 epidemic continues to affect rural and periurban environments. For example, it will be important to evaluate the impact of new government policies, such as the ending of temporary increases in noncontributory grants. Second, these behavior-related data can be linked to COVID-19 symptoms and individual and population health outcomes to evaluate how risk perceptions and reactions are associated with health outcomes. Third, a more in-depth analysis of how household members’ historical and current age, gender, employment status, and migration composition, as well as preexisting comorbidities, affect the impacts of COVID-19 and NPIs will help identify those who are most in need of support during such crises. The ongoing SAPRIN COVID-19 surveillance program will enable the longitudinal measurement of these factors throughout the epidemic’s course.

### Strengths and Limitations

This study has limitations. As with all observational studies, the generalizability of our results to those outside our study population—in this case, households in rural and periurban areas of eastern South Africa—is uncertain. This concern was tempered by our ability to compare and combine data across multiple sites and compare our results to those of other studies on the COVID-19 epidemic’s impact in South Africa and Sub-Saharan African. Additionally, while household cellphone ownership was high, there was evidence that lower-wealth households in these areas were somewhat less likely to participate in the survey. Although nonrandom response may have affected prevalence measures, it should have very limited scope to affect the trend measures we focused on. Further, our data were self-reported and thus represented perceived needs and impacts, and changes in reported behavior may have reflected desirability biases. However, even with such biases, our findings provide insight into the perceptions and lived experiences of these communities. Comparing our findings to digital data sources can help alleviate such biases. Finally, we did not have data on identical questions from the pre-epidemic period; however, we were able to include similar information on many topics from earlier surveillance studies.

This study also has several strengths, including a clearly defined sampling base, high response rates, low attrition rates, frequent follow-up, and linkages to pre-epidemic household data. Although we were not able to interview the same people in every survey wave, our longitudinal household cohort design, which allowed for repeated interviews with the same households over multiple months, reduced the risk of confounding by time-invariant household factors that could have arisen if we had used multiple cross-sectional surveys. Many of these benefits arise from the nature of the existing SAPRIN surveillance infrastructure, which reinforces the importance of long-term, population-based surveillance systems that collect social, demographic, and health data. This study demonstrates that surveillance systems can be rapidly repurposed to respond to emergency health needs, including (1) rapid pathogen data acquisition; (2) the identification of susceptible populations; (3) the assessment of behavioral and biomedical interventions; and (4) the development of mitigation strategies [49].

SAPRIN nodes have been working with their local communities for 20-28 years. Such long-term engagement promotes deep understanding and community buy-in, which in turn enables rapid implementation and sustained, high-intensity follow-ups with minimal dropout. The network nature of SAPRIN also allowed each node to flexibly implement an overarching protocol. Furthermore, the use of telephonic call centers at each node allowed for rapid survey rollout that was based on previously provided informed consent for personal calls and substantially reduced the risk of SARS-CoV-2 infection among study staff and research participants. Since these call centers employed locally recruited staff, we were able to reach population segments that web-based surveys (in a country with rural areas that have limited access to internet) and even random-digit dialing approaches (in a country with 11 official spoken languages and numerous dialects) struggle to capture. Additionally, we could link self-response survey data to other data sources within the SAPRIN databases. These include the previously collected sociodemographic data used in this study and biological samples that were collected as part of the COVID-19 surveillance project. SAPRIN data can also be linked to data on public sector health care use and laboratory test results through memoranda of understanding with government departments. SAPRIN’s ongoing expansion will also allow comparisons with well-characterized urban sites to be made in the future.

### Conclusion

South Africans in three rural and periurban areas were largely willing and able to comply with national government regulations and recommendations regarding social interaction and other risk behaviors related to COVID-19, despite limited resources and the substantial economic need to travel. This rapid uptake of preventative behaviors reflects the clarity of government messages and the population’s willingness to comply with such measures, even in settings where enforcement measures were limited. Even as official NPIs were relaxed, the arrival of the epidemic in local areas led to further self-imposed behavioral restrictions, several of which led to difficulties in accessing health care. However, the economic and mental health effects of NPIs continued to decline as the measures were eased. Our findings highlight the importance of monitoring the possibly deleterious secondary impacts of NPIs in epidemic situations. Our results reinforce the principle that NPIs should be adjusted based on epidemic cycles and show that mitigation measures will be required to combat anticipated and unanticipated secondary impacts. All of these factors should be considered when setting, adjusting, and relaxing NPIs in low-income settings, especially as urgently established national policies give way to differentiated, decentralized approaches across diverse subnational environments.

## Appendix

Multimedia Appendix 1 Supplementary materials.

## Abbreviations

AHRI: Africa Health Research Institute
CRAM: Coronavirus Rapid Mobile Survey
DIMAMO: Dikgale-Mamabolo-Mothiba
HDSS: Health and Demographic Surveillance System
GAD-2: Generalized Anxiety Disorder 2-item
MRC: Medical Research Council
NIDS: National Income Dynamics Study
NPI: nonpharmaceutical intervention
PHQ-2: Patient Health Questionnaire 2-item
REC: Research Ethics Committee
SAPRIN: South African Population Research Infrastructure Network
