# Supplementary material for: Protective Behaviors and Secondary Harms Resulting From Nonpharmaceutical Interventions During the COVID-19 Epidemic in South Africa: Multisite, Prospective Longitudinal Study
Source: JMIR Public Health Surveill. 2021 May 13;7(5):e26073. doi: 10.2196/26073 (PMC8121138; doi:10.2196/26073)
Supplement: Multimedia Appendix 1 [file publichealth_v7i5e26073_app1.docx]

# Supplementary Material

**Title**: Protective behaviours and secondary harms from non-pharmaceutical interventions during the COVID-19 epidemic in South Africa: a multisite prospective longitudinal study

Material S1: COVID-19 household interview

| **Variable** | **Question** | **Notes** |
| --- | --- | --- |
| **Household Level Questions** | | |
|  | **Actions in response to COVID-19** | |
| ar01 | Have you heard of COVID-19 or Coronavirus | Yes; No; Don't Know |
| ar02 ar01==Yes | Have you or members of your household changed anything you do as a result of hearing about COVID-19 or Coronavirus? | Yes; No; Don't Know |
| ar03 ar02==Yes | What things have you or your household changed as a result of hearing about COVID-19 or Coronavirus? (mark all that apply) | Washed hands more often; Avoiding crowded areas; Avoided social events; Avoided taking taxis; Avoided going out; Avoided going to work; Avoided travelling long distances; Using face masks; Wearing gloves; Using hand sanitizer; Other |
| ar04 ar03==Other | Specify other |  |
| xx01 | How much do you feel you know about the COVID-19 pandemic? | Less than I should know; A little, but not enough; Enough; A little more than most people; I am up to date on the latest research |
| **Household Members and Visitors** | | |
| hv01 | Thinking of the whole of yesterday, did anyone visit your home who did not sleep in the house the previous night? | Yes; No; Don't Know |
| hv02 hv01==1 | How many such visitors were there yesterday? If you are not sure, please make your best guess. |  |
| hs01 | To your knowledge does everyone in your household who usually takes a daily medication had access to all necessary doses over the last week? | Yes; No; Don't Know |
| **Household Impact of COVID-19** | | |
| hi01 | Over the past seven days, have you or any member of your household wanted to access healthcare but have been unable to do so? | Yes; No; Don't Know |
| hi03 | Over the past seven days, has your family been able to get all food and other household necessities they need? | Yes; No; Don't Know |
| hi04 | Over the past seven days, have COVID-19 laws/regulations/rules affected the ability of you or your household to earn money? | Yes; No; Don't Know |
| hi05 hi04==Yes | The ability of you or your household to earn money over the past seven days? Select all that apply | Someone in the household has lost a job; Someone in the household is on unpaid leave; Other |
| hi06 hi04==Yes | What are you planning to do to as a result of this financial hardship? Select all that apply | Not pay bills that are due; Take out a loan; Skip meals; Other |
| hi07 | Over the past seven days, have you had access to soap and water at home for hand hygiene? | Always; Sometimes; Never |
| PHQ2_1 | Over the past seven days, how often have you been bothered by having little interest or pleasure in doing things? | Not at all; Several days; More than half the days; Nearly every day |
| PHQ2_2 | Over the past seven days, how often have you been bothered by feeling down, depressed or hopeless? | Not at all; Several days; More than half the days; Nearly every day |
| GAD2_1 | Over the past seven days, how often have you been bothered by feeling nervous, anxious or on edge? | Not at all; Several days; More than half the days; Nearly every day |
| GAD2_2 | Over the past seven days, how often have you been bothered by not be able to stop or control worrying. | Not at all; Several days; More than half the days; Nearly every day |

| **Travel and Movement** | | |
| --- | --- | --- |
| tm01 | Over the past seven days, have %rostertitle% left your home? | Yes; No; Don't Know |
| tm02 tm01==Yes | Over the past seven days, have %rostertitle% left your village/suburb or isigodi? | Yes; No; Don't Know |
| tm03 tm02==Yes | Over the past seven days, have %rostertitle% travelled to your local town (e.g. Mtubatuba, KwaMsane, Hlabisa, Hluhluwe, St Lucia) | Yes; No; Don't Know |
| tm04 tm02==Yes | Over the past seven days, have %rostertitle% travelled beyond the subdistrict (e.g. Mtubatuba municipality)? | Yes; No; Don't Know |
| tm05 | Why did %rostertitle% leave your home? (multi-select) | To get food/medication; To go to work; To visit friends or family; To get exercise; To provide care to a vulnerable person; Other |
| tm06 | Other, specify? |  |

Figure S1: Map of SAPRIN nodes as of September 2020


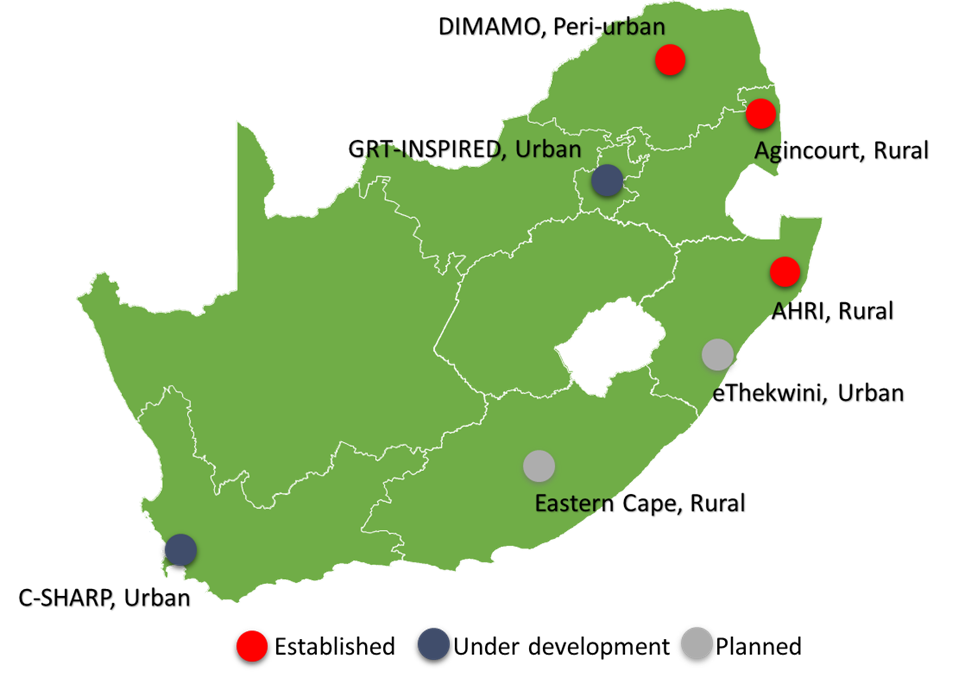


Figure S2: Sampling and participation patterns by SAPRIN node and interview round in South Africa April-August 2020

|  |  | Number of households | | | |  | | Wave | | | | | | | | | | | | |
| --- | --- | --- | --- | --- | --- | --- | --- | --- | --- | --- | --- | --- | --- | --- | --- | --- | --- | --- | --- | --- |
|  | Cohort | Sampled | No contact information | Dropped after  no wave 1 participation | Ever  interviewed |  | 1 | 2 | 3 | 4 | 5 | 6 | 7 | 8 | 9 | 10 | 11 | 12 | 13 | 14 |
| AHRI  (KwaZulu-Natal) |  |  |  |  |  |  |  |  |  |  |  |  |  |  |  |  |  |  |  |  |
|  | 1 | 250 |  |  | 238 |  |  |  |  |  |  |  |  |  |  |  |  |  |  |  |
|  | 2 | 250 |  |  | 237 |  |  |  |  |  |  |  |  |  |  |  |  |  |  |  |
|  | 3 | 250 |  |  | 241 |  |  |  |  |  |  |  |  |  |  |  |  |  |  |  |
|  | 4 | 250 |  |  | 224 |  |  |  |  |  |  |  |  |  |  |  |  |  |  |  |
|  | 5 | 250 |  |  | 236 |  |  |  |  |  |  |  |  |  |  |  |  |  |  |  |
|  | 6 | 250 |  |  | 220 |  |  |  |  |  |  |  |  |  |  |  |  |  |  |  |
|  | 7 | 250 |  |  | 212 |  |  |  |  |  |  |  |  |  |  |  |  |  |  |  |
| Agincourt (Mpumalanga) |  |  |  |  |  |  |  |  |  |  |  |  |  |  |  |  |  |  |  |  |
|  | 1 | 1726 | 477 | 589 | 660 |  |  |  |  |  |  |  |  |  |  |  |  |  |  |  |
|  | 2 | 249 |  |  | 249 |  |  |  |  |  |  |  |  |  |  |  |  |  |  |  |
|  | 3 | 250 |  |  | 250 |  |  |  |  |  |  |  |  |  |  |  |  |  |  |  |
|  | 4 | 247 |  |  | 234 |  |  |  |  |  |  |  |  |  |  |  |  |  |  |  |
|  | 5 | 251 |  |  | 204 |  |  |  |  |  |  |  |  |  |  |  |  |  |  |  |
|  | 6 | 252 |  |  | 200 |  |  |  |  |  |  |  |  |  |  |  |  |  |  |  |
| DIMAMO (Limpopo) |  |  |  |  |  |  |  |  |  |  |  |  |  |  |  |  |  |  |  |  |
|  | 1 | 333 | 31 |  | 288 |  |  |  |  |  |  |  |  |  |  |  |  |  |  |  |
|  | 2 | 333 | 43 |  | 274 |  |  |  |  |  |  |  |  |  |  |  |  |  |  |  |
|  | 3 | 334 | 44 |  | 273 |  |  |  |  |  |  |  |  |  |  |  |  |  |  |  |
|  | 4 | 333 | 39 |  | 243 |  |  |  |  |  |  |  |  |  |  |  |  |  |  |  |
|  | 5 | 333 | 45 |  | 229 |  |  |  |  |  |  |  |  |  |  |  |  |  |  |  |
|  | 6 | 333 | 42 |  | 226 |  |  |  |  |  |  |  |  |  |  |  |  |  |  |  |
|  | 7 | 333 | 53 |  | 182 |  |  |  |  |  |  |  |  |  |  |  |  |  |  |  |
|  |  |  |  |  |  |  |  |  |  |  |  |  |  |  |  |  |  |  |  |  |
| Total |  | 7057 | 774 |  | 5120 |  |  |  |  |  |  |  |  |  |  |  |  |  |  |  |

Table S1: Response patterns for eligible households by SAPRIN node and interview round in South Africa April-August 2020

| **Node** | **Wave** |  | **Phone out of order/wrong number** | |  | **Phone not  answered** | | |  | | **Refused call** | |  | | **Successful** | | |  | | **Total** | |  |
| --- | --- | --- | --- | --- | --- | --- | --- | --- | --- | --- | --- | --- | --- | --- | --- | --- | --- | --- | --- | --- | --- | --- |
| Agincourt | 1 |  | 257 | (14.6%) |  | 191 | (10.9%) |  | | 154 | | (8.8%) | |  | | 1,159 | (65.8%) | |  | | 1,761 | |
| (Mpumalanga) | 2 |  | 21 | (1.9%) |  | 59 | (5.3%) |  | | 29 | | (2.6%) | |  | | 1,013 | (90.3%) | |  | | 1,122 | |
|  | 3 |  | 36 | (3.2%) |  | 94 | (8.4%) |  | | 49 | | (4.4%) | |  | | 944 | (84.1%) | |  | | 1,123 | |
|  | 4 |  | 59 | (5.1%) |  | 72 | (6.2%) |  | | 24 | | (2.1%) | |  | | 1,004 | (86.6%) | |  | | 1,159 | |
|  | 5 |  | 89 | (7.7%) |  | 89 | (7.7%) |  | | 30 | | (2.6%) | |  | | 951 | (82.1%) | |  | | 1,159 | |
|  | 6 |  | 136 | (11.7%) |  | 98 | (8.5%) |  | | 51 | | (4.4%) | |  | | 874 | (75.4%) | |  | | 1,159 | |
|  | 7 |  | 122 | (10.5%) |  | 100 | (8.6%) |  | | 41 | | (3.5%) | |  | | 896 | (77.3%) | |  | | 1,159 | |
|  | 8 |  | 52 | (6.9%) |  | 40 | (5.3%) |  | | 26 | | (3.5%) | |  | | 632 | (84.3%) | |  | | 750 | |
|  | 9 |  | 44 | (5.9%) |  | 42 | (5.6%) |  | | 25 | | (3.3%) | |  | | 639 | (85.2%) | |  | | 750 | |
|  | 10 |  | 85 | (11.3%) |  | 81 | (10.8%) |  | | 34 | | (4.5%) | |  | | 550 | (73.3%) | |  | | 750 | |
|  | 11 |  | 145 | (19.3%) |  | 141 | (18.8%) |  | | 37 | | (4.9%) | |  | | 427 | (56.9%) | |  | | 750 | |
|  | 12 |  | 96 | (12.8%) |  | 97 | (12.9%) |  | | 38 | | (5.1%) | |  | | 519 | (69.2%) | |  | | 750 | |
|  |  |  |  |  |  |  |  |  | |  | |  | |  | |  |  | |  | |  | |
| AHRI | 1 |  | 69 | (9.2%) |  | 69 | (9.2%) |  | | 8 | | (1.1%) | |  | | 604 | (80.5%) | |  | | 750 | |
| (KwaZulu-Natal) | 2 |  | 63 | (8.4%) |  | 141 | (18.8%) |  | | 16 | | (2.1%) | |  | | 530 | (70.7%) | |  | | 750 | |
|  | 3 |  | 48 | (6.4%) |  | 186 | (24.8%) |  | | 24 | | (3.2%) | |  | | 492 | (65.6%) | |  | | 750 | |
|  | 4 |  | 69 | (9.2%) |  | 92 | (12.3%) |  | | 20 | | (2.7%) | |  | | 569 | (75.9%) | |  | | 750 | |
|  | 5 |  | 77 | (10.3%) |  | 140 | (18.7%) |  | | 17 | | (2.3%) | |  | | 516 | (68.8%) | |  | | 750 | |
|  | 6 |  | 52 | (6.9%) |  | 159 | (21.2%) |  | | 8 | | (1.1%) | |  | | 531 | (70.8%) | |  | | 750 | |
|  | 7 |  | 45 | (6.0%) |  | 204 | (27.2%) |  | | 10 | | (1.3%) | |  | | 491 | (65.5%) | |  | | 750 | |
|  | 8 |  | 48 | (6.4%) |  | 243 | (32.4%) |  | | 14 | | (1.9%) | |  | | 445 | (59.3%) | |  | | 750 | |
|  | 9 |  | 104 | (13.9%) |  | 211 | (28.1%) |  | | 8 | | (1.1%) | |  | | 427 | (56.9%) | |  | | 750 | |
|  | 10 |  | 10 | (1.3%) |  | 251 | (33.5%) |  | | 11 | | (1.5%) | |  | | 478 | (63.7%) | |  | | 750 | |
|  | 11 |  | 97 | (12.9%) |  | 154 | (20.5%) |  | | 10 | | (1.3%) | |  | | 489 | (65.2%) | |  | | 750 | |
|  | 12 |  | 23 | (3.1%) |  | 237 | (31.6%) |  | | 5 | | (0.7%) | |  | | 485 | (64.7%) | |  | | 750 | |
|  | 13 |  | 54 | (7.2%) |  | 138 | (18.4%) |  | | 14 | | (1.9%) | |  | | 544 | (72.5%) | |  | | 750 | |
|  | 14 |  | 77 | (10.3%) |  | 168 | (22.4%) |  | | 18 | | (2.4%) | |  | | 487 | (64.9%) | |  | | 750 | |
|  |  |  |  |  |  |  |  |  | |  | |  | |  | |  |  | |  | |  | |
| DIMAMO | 1 |  | 82 | (9.3%) |  | 63 | (7.2%) |  | | 31 | | (3.5%) | |  | | 705 | (80.0%) | |  | | 881 | |
| (Limpopo) | 2 |  | 87 | (9.9%) |  | 78 | (8.8%) |  | | 32 | | (3.6%) | |  | | 677 | (76.8%) | |  | | 874 | |
|  | 3 |  | 67 | (7.6%) |  | 86 | (9.8%) |  | | 64 | | (7.3%) | |  | | 644 | (73.0%) | |  | | 861 | |
|  | 4 |  | 77 | (8.7%) |  | 61 | (6.9%) |  | | 70 | | (7.9%) | |  | | 603 | (68.4%) | |  | | 811 | |
|  | 5 |  | 71 | (8.1%) |  | 61 | (6.9%) |  | | 38 | | (4.3%) | |  | | 571 | (64.7%) | |  | | 741 | |
|  | 6 |  | 86 | (9.8%) |  | 72 | (8.2%) |  | | 31 | | (3.5%) | |  | | 514 | (58.3%) | |  | | 703 | |
|  | 7 |  | 72 | (8.2%) |  | 123 | (14.1%) |  | | 46 | | (5.3%) | |  | | 499 | (57.1%) | |  | | 740 | |
|  | 8 |  | 102 | (11.8%) |  | 76 | (8.8%) |  | | 67 | | (7.7%) | |  | | 557 | (64.2%) | |  | | 802 | |
|  | 9 |  | 132 | (15.2%) |  | 45 | (5.2%) |  | | 52 | | (6.0%) | |  | | 576 | (66.4%) | |  | | 805 | |
|  | 10 |  | 121 | (13.9%) |  | 47 | (5.4%) |  | | 36 | | (4.2%) | |  | | 549 | (63.3%) | |  | | 753 | |
|  | 11 |  | 127 | (14.7%) |  | 95 | (11.0%) |  | | 54 | | (6.2%) | |  | | 504 | (58.2%) | |  | | 780 | |
|  |  |  |  |  |  |  |  |  | |  | |  | |  | |  |  | |  | |  | |
| Total |  |  | 3,002 | (9.2%) |  | 4,304 | (13.2%) |  | | 1,242 | | (3.8%) | |  | | 23,095 | (71.0%) | |  | | 31,643 | |

All interviews up to end of 2020. Households sampled with no telephone numbers are excluded. At Agincourt: 465 of 2,225 (wave 1); at DIMAMO: 119 of 1,000 (wave 1); 39 of 333 (wave 7); 45 of 333 (wave 8); 42 of 333 (wave 9); 53 of 333 (wave 10). From wave 2, Agincourt only sought to reach the 1159 households that participated in wave 1, although in waves 2 and 3 some households were unintentionally not contacted. Households refusing at each round were not followed up subsequently at DIMAMO.

Table S2: Comparison of characteristics of participating and non-participating SAPRIN households in South Africa April-August 2020

|  |  |  | **Participation** | | |
| --- | --- | --- | --- | --- | --- |
|  | **Total** |  | **No** | **Yes** | **p-value** |
| N | **6,287** |  | **1,167** | **5,120** |  |
| Highest educational attainment |  |  |  |  | < 0.001 |
| Less than complete secondary | 18.2% |  | 21.2% | 17.5% |  |
| Complete secondary | 49.0% |  | 54.9% | 47.7% |  |
| Diploma/certificate/degree | 14.6% |  | 10.4% | 15.6% |  |
| Missing | 18.2% |  | 13.5% | 19.3% |  |
| Node-specific household wealth quintile |  |  |  |  | 0.83 |
| Lowest | 11.5% |  | 10.9% | 11.7% |  |
| Second lowest | 15.1% |  | 14.7% | 15.2% |  |
| Middle | 17.4% |  | 17.4% | 17.4% |  |
| Second highest | 19.0% |  | 18.6% | 19.0% |  |
| Highest | 20.3% |  | 21.3% | 20.1% |  |
| Missing | 16.7% |  | 17.1% | 16.6% |  |
| Household size in 2020 ^†^ | 5 (3, 8) |  |  | 5 (3, 8) |  |
| Number of children | 2 (1, 3) |  |  | 2 (1, 3) |  |
| Number of working age adults | 3 (2, 4) |  |  | 3 (2, 4) |  |
| Number of people aged over 60 | 0 (0, 1) |  |  | 0 (0, 1) |  |
| Number of full/part-time employed people | 1 (0, 2) |  | 1 (0, 2) | 1 (0, 2) | 0.007 * |
| Number of pension grant receivers | 0 (0, 1) |  | 0 (0, 1) | 0 (0, 1) | 0.57 |
| Number of non-pension grant receivers | 1 (0, 3) |  | 1 (0, 3) | 1 (0, 3) | 0.44 |

Data are medians (IQR) for continuous measures, and percent for categorical measures. Percentages are of all households for ‘participation’ and of all non-missing values for ‘node’ columns. P-values are for difference between groups within characteristics using $\chi^{2}$ tests for categorical variables and Wilcoxon rank-sum for continuous variables. ^†^Household sizes in 2020 only captured for participating households. ^*^ Participating households have more employed individuals.

Table S3: Proportions of households affirming questions by node and month of interview

| **AHRI** |  |  |  |  |  |  |  |  |  |  |
| --- | --- | --- | --- | --- | --- | --- | --- | --- | --- | --- |
|  | **April** | | **May** | | **June** | | **July** | | **August** | |
| Enough knowledge | 48.3 | [39.3, 57.3] | 56.3 | [52.6, 60.1] | 68.6 | [65.5, 71.7] | 67.6 | [64.6, 70.5] | 85.3 | [83.2, 87.4] |
| Any visitors | 7.6 | [2.8, 12.4] | 10.4 | [8.1, 12.7] | 7.8 | [6.0, 9.6] | 4.7 | [3.4, 6.0] | 3.7 | [2.6, 4.9] |
| Left home | 28.2 | [20.1, 36.4] | 64.7 | [61.0, 68.3] | 76.2 | [73.3, 79.0] | 31.8 | [28.9, 34.8] | 18.0 | [15.7, 20.3] |
| Avoid crowds | 88.8 | [83.1, 94.5] | 87.0 | [84.5, 89.6] | 79.2 | [76.5, 82.0] | 51.9 | [48.7, 55.1] | 63.7 | [60.7, 66.6] |
| Avoid transport | 42.2 | [33.3, 51.2] | 49.9 | [46.1, 53.7] | 42.0 | [38.7, 45.3] | 33.2 | [30.2, 36.2] | 27.1 | [24.4, 29.8] |
| Use facemask | 20.7 | [13.3, 28.1] | 89.1 | [86.8, 91.5] | 98.0 | [97.0, 98.9] | 90.6 | [88.8, 92.5] | 96.5 | [95.3, 97.6] |
| Unable access medication | 45.8 | [36.8, 54.8] | 36.1 | [32.5, 39.8] | 37.4 | [34.2, 40.7] | 64.9 | [61.9, 67.9] | 60.1 | [57.1, 63.0] |
| Unmet healthcare need | 11.0 | [5.4, 16.7] | 9.3 | [7.1, 11.6] | 8.2 | [6.3, 10.0] | 11.9 | [9.9, 14.0] | 6.4 | [4.9, 7.8] |
| Lost earnings | 50.0 | [41.0, 59.0] | 30.9 | [27.4, 34.4] | 22.3 | [19.5, 25.1] | 19.1 | [16.6, 21.5] | 15.3 | [13.1, 17.4] |
| PHQ-2 | 16.9 | [10.2, 23.7] | 11.9 | [9.4, 14.4] | 4.6 | [3.2, 6.0] | 8.1 | [6.4, 9.8] | 4.8 | [3.5, 6.1] |
| GAD-2 | 28.0 | [19.9, 36.1] | 14.0 | [11.4, 16.6] | 12.1 | [9.9, 14.3] | 1.8 | [1.0, 2.7] | 2.3 | [1.4, 3.3] |
|  |  |  |  |  |  |  |  |  |  |  |
| **Agincourt** |  |  |  |  |  |  |  |  |  |  |
|  | **April** | | **May** | | **June** | | **July** | | **August** | |
| Enough knowledge |  |  | 52.5 | [49.5, 55.6] | 71.4 | [68.6, 74.2] | 84.6 | [83.0, 86.3] | 90.1 | [88.6, 91.6] |
| Any visitors |  |  | 12.9 | [10.9, 15.0] | 8.8 | [7.1, 10.6] | 11.3 | [9.9, 12.8] | 11.0 | [9.5, 12.6] |
| Left home |  |  | 65.3 | [62.4, 68.2] | 82.3 | [79.9, 84.7] | 86.3 | [84.7, 87.9] | 87.1 | [85.4, 88.8] |
| Avoid crowds |  |  | 28.3 | [25.6, 31.1] | 63.6 | [60.6, 66.5] | 61.0 | [58.8, 63.2] | 69.7 | [67.4, 71.9] |
| Avoid transport |  |  | 20.5 | [18.0, 23.0] | 54.0 | [51.0, 57.1] | 48.7 | [46.4, 51.0] | 53.4 | [51.0, 55.9] |
| Use facemask |  |  | 63.4 | [60.5, 66.4] | 93.8 | [92.3, 95.2] | 95.5 | [94.6, 96.5] | 97.7 | [96.9, 98.4] |
| Unable access medication |  |  | 51.9 | [48.8, 54.9] | 57.1 | [54.0, 60.1] | 51.3 | [49.0, 53.5] | 44.4 | [41.9, 46.9] |
| Unmet healthcare need |  |  | 11.2 | [9.3, 13.1] | 3.5 | [2.4, 4.7] | 2.4 | [1.7, 3.1] | 2.4 | [1.6, 3.1] |
| Lost earnings |  |  | 27.4 | [24.6, 30.1] | 11.4 | [9.4, 13.3] | 14.4 | [12.8, 16.0] | 16.3 | [14.5, 18.1] |
| PHQ-2 |  |  | 5.2 | [3.8, 6.5] | 1.6 | [0.8, 2.4] | 4.0 | [3.1, 4.9] | 2.8 | [2.0, 3.6] |
| GAD-2 |  |  | 5.0 | [3.6, 6.3] | 1.5 | [0.7, 2.2] | 4.0 | [3.1, 4.9] | 1.9 | [1.2, 2.5] |
|  |  |  |  |  |  |  |  |  |  |  |
| **DIMAMO** |  |  |  |  |  |  |  |  |  |  |
|  | **April** | | **May** | | **June** | | **July** | | **August** | |
| Enough knowledge |  |  |  |  |  |  | 59.6 | [56.0, 63.2] | 75.5 | [72.9, 78.0] |
| Any visitors |  |  |  |  |  |  | 4.0 | [2.5, 5.4] | 4.5 | [3.2, 5.7] |
| Left home |  |  |  |  |  |  | 83.0 | [80.2, 85.7] | 87.6 | [85.6, 89.6] |
| Avoid crowds |  |  |  |  |  |  | 93.3 | [91.4, 95.2] | 93.8 | [92.3, 95.3] |
| Avoid transport |  |  |  |  |  |  | 76.8 | [73.6, 80.0] | 64.4 | [61.5, 67.3] |
| Use facemask |  |  |  |  |  |  | 97.0 | [95.7, 98.3] | 98.6 | [97.9, 99.3] |
| Unable access medication |  |  |  |  |  |  | 66.0 | [62.5, 69.5] | 62.9 | [60.0, 65.8] |
| Unmet healthcare need |  |  |  |  |  |  | 10.2 | [8.0, 12.4] | 9.0 | [7.3, 10.8] |
| Lost earnings |  |  |  |  |  |  | 13.8 | [11.2, 16.3] | 6.9 | [5.4, 8.4] |
| PHQ-2 |  |  |  |  |  |  | 39.6 | [36.0, 43.2] | 21.6 | [19.2, 24.1] |
| GAD-2 |  |  |  |  |  |  | 53.5 | [49.8, 57.2] | 33.0 | [30.2, 35.8] |
|  |  |  |  |  |  |  |  |  |  |  |
|  |  |  |  |  |  |  |  |  |  |  |
| **Total** |  |  |  |  |  |  |  |  |  |  |
|  | **April** | | **May** | | **June** | | **July** | | **August** | |
| Enough knowledge | 48.3 | [39.3, 57.3] | 54.0 | [51.6, 56.4] | 70.1 | [68.0, 72.2] | 74.8 | [73.4, 76.3] | 84.5 | [83.3, 85.7] |
| Any visitors | 7.6 | [2.8, 12.4] | 11.9 | [10.4, 13.5] | 8.4 | [7.1, 9.6] | 8.0 | [7.1, 8.9] | 7.0 | [6.2, 7.9] |
| Left home | 28.2 | [20.1, 36.4] | 65.0 | [62.8, 67.3] | 79.5 | [77.6, 81.3] | 70.3 | [68.8, 71.8] | 67.2 | [65.7, 68.7] |
| Avoid crowds | 88.8 | [83.1, 94.5] | 51.3 | [48.9, 53.6] | 70.6 | [68.6, 72.7] | 64.8 | [63.2, 66.4] | 74.8 | [73.4, 76.2] |
| Avoid transport | 42.2 | [33.3, 51.2] | 32.0 | [29.8, 34.2] | 48.6 | [46.3, 50.8] | 50.0 | [48.3, 51.7] | 49.0 | [47.4, 50.6] |
| Use facemask | 20.7 | [13.3, 28.1] | 73.5 | [71.4, 75.6] | 95.7 | [94.7, 96.6] | 94.5 | [93.7, 95.2] | 97.6 | [97.1, 98.1] |
| Unable access medication | 45.8 | [36.8, 54.8] | 45.7 | [43.3, 48.1] | 48.2 | [45.9, 50.4] | 58.0 | [56.4, 59.6] | 54.3 | [52.7, 55.9] |
| Unmet healthcare need | 11.0 | [5.4, 16.7] | 10.5 | [9.0, 11.9] | 5.6 | [4.6, 6.7] | 6.6 | [5.8, 7.4] | 5.5 | [4.7, 6.2] |
| Lost earnings | 50.0 | [41.0, 59.0] | 28.7 | [26.6, 30.9] | 16.3 | [14.6, 18.0] | 15.6 | [14.4, 16.8] | 13.3 | [12.2, 14.4] |
| PHQ-2 | 16.9 | [10.2, 23.7] | 7.8 | [6.5, 9.1] | 3.0 | [2.2, 3.7] | 12.3 | [11.2, 13.4] | 8.8 | [7.9, 9.8] |
| GAD-2 | 28.0 | [19.9, 36.1] | 8.5 | [7.2, 9.8] | 6.3 | [5.2, 7.4] | 13.3 | [12.2, 14.4] | 11.0 | [10.0, 12.0] |

| **AHRI** |  |  |  |  |  |  |  |  |
| --- | --- | --- | --- | --- | --- | --- | --- | --- |
|  | **September** | | **October** | | **November** | | **December** | |
| Enough knowledge | 85.6 | [83.4, 87.8] | 84.6 | [82.5, 86.7] | 90.0 | [88.2, 91.8] | 95.9 | [93.3, 98.4] |
| Any visitors | 4.4 | [3.1, 5.7] | 5.1 | [3.8, 6.4] | 5.9 | [4.5, 7.3] | 4.1 | [1.6, 6.7] |
| Left home | 25.3 | [22.5, 28.0] | 35.4 | [32.6, 38.1] | 31.0 | [28.2, 33.7] | 41.5 | [35.3, 47.7] |
| Avoid crowds | 72.4 | [69.5, 75.2] | 74.9 | [72.3, 77.5] | 77.0 | [74.4, 79.5] | 69.7 | [63.6, 75.7] |
| Avoid transport | 45.9 | [42.7, 49.1] | 51.7 | [48.7, 54.7] | 48.9 | [45.8, 51.9] | 27.6 | [21.7, 33.5] |
| Use facemask | 97.9 | [97.0, 98.8] | 99.6 | [99.2, 100.0] | 98.6 | [97.9, 99.3] | 95.5 | [92.7, 98.2] |
| Unable access medication | 22.4 | [19.8, 25.1] | 34.0 | [31.2, 36.7] | 38.5 | [35.5, 41.4] | 46.5 | [40.2, 52.8] |
| Unmet healthcare need | 6.6 | [5.0, 8.1] | 2.3 | [1.4, 3.2] | 10.5 | [8.7, 12.3] | 9.1 | [5.5, 12.8] |
| Lost earnings | 12.0 | [9.9, 14.1] | 6.2 | [4.8, 7.6] | 6.5 | [5.0, 7.9] | 5.8 | [2.9, 8.8] |
| PHQ-2 | 8.1 | [6.4, 9.9] | 6.5 | [5.1, 8.0] | 11.9 | [10.0, 13.9] | 7.5 | [4.1, 10.8] |
| GAD-2 | 4.4 | [3.1, 5.7] | 0.9 | [0.3, 1.4] | 8.2 | [6.5, 9.8] | 9.1 | [5.5, 12.8] |
|  |  |  |  |  |  |  |  |  |
| **Agincourt** |  |  |  |  |  |  |  |  |
|  | **September** | | **October** | | **November** | | **December** | |
| Enough knowledge | 95.0 | [93.8, 96.3] | 99.3 | [98.7, 99.9] | 93.7 | [92.3, 95.2] | 97.8 | [96.9, 98.7] |
| Any visitors | 10.6 | [8.8, 12.3] | 12.0 | [9.8, 14.2] | 12.8 | [10.9, 14.8] | 6.5 | [4.9, 8.0] |
| Left home | 92.2 | [90.7, 93.8] | 92.8 | [91.1, 94.6] | 92.3 | [90.7, 93.9] | 78.8 | [76.2, 81.4] |
| Avoid crowds | 69.5 | [66.8, 72.1] | 48.0 | [44.6, 51.4] | 42.6 | [39.6, 45.5] | 48.3 | [45.1, 51.4] |
| Avoid transport | 50.3 | [47.5, 53.2] | 32.5 | [29.4, 35.7] | 43.8 | [40.8, 46.7] | 42.1 | [39.0, 45.3] |
| Use facemask | 98.7 | [98.1, 99.4] | 98.9 | [98.2, 99.6] | 98.8 | [98.2, 99.5] | 99.1 | [98.4, 99.7] |
| Unable access medication | 45.2 | [42.3, 48.0] | 48.1 | [44.7, 51.5] | 52.0 | [49.0, 55.0] | 54.0 | [50.8, 57.1] |
| Unmet healthcare need | 0.8 | [0.3, 1.4] | 1.6 | [0.7, 2.4] | 0.9 | [0.3, 1.5] | 1.0 | [0.4, 1.7] |
| Lost earnings | 12.1 | [10.3, 14.0] | 10.7 | [8.6, 12.8] | 8.8 | [7.1, 10.4] | 11.6 | [9.6, 13.6] |
| PHQ-2 | 1.4 | [0.8, 2.1] | 0.2 | [-0.1, 0.6] | 0.8 | [0.3, 1.4] | 8.4 | [6.6, 10.1] |
| GAD-2 | 0.6 | [0.2, 1.0] | 0.4 | [0.0, 0.8] | 0.8 | [0.3, 1.4] | 9.2 | [7.4, 11.0] |
|  |  |  |  |  |  |  |  |  |
| **DIMAMO** |  |  |  |  |  |  |  |  |
|  | **September** | | **October** | | **November** | | **December** | |
| Enough knowledge | 77.6 | [75.3, 79.8] | 77.1 | [74.6, 79.5] | 82.8 | [80.7, 84.9] | 85.4 | [83.2, 87.7] |
| Any visitors | 4.9 | [3.7, 6.0] | 10.4 | [8.6, 12.2] | 11.5 | [9.8, 13.3] | 7.6 | [6.0, 9.3] |
| Left home | 96.4 | [95.4, 97.4] | 95.9 | [94.7, 97.0] | 99.2 | [98.7, 99.7] | 99.6 | [99.2, 100.0] |
| Avoid crowds | 93.0 | [91.6, 94.4] | 85.5 | [83.4, 87.5] | 81.5 | [79.3, 83.7] | 74.8 | [72.1, 77.6] |
| Avoid transport | 72.0 | [69.6, 74.5] | 88.4 | [86.5, 90.3] | 91.2 | [89.6, 92.8] | 89.6 | [87.7, 91.6] |
| Use facemask | 98.5 | [97.9, 99.2] | 99.5 | [99.0, 99.9] | 99.4 | [99.0, 99.8] | 99.6 | [99.2, 100.0] |
| Unable access medication | 69.4 | [66.9, 71.9] | 77.3 | [74.9, 79.8] | 70.2 | [67.6, 72.8] | 63.1 | [60.0, 66.1] |
| Unmet healthcare need | 11.0 | [9.3, 12.6] | 9.0 | [7.3, 10.7] | 13.0 | [11.1, 14.9] | 14.3 | [12.1, 16.6] |
| Lost earnings | 3.2 | [2.2, 4.1] | 1.3 | [0.7, 2.0] | 0.7 | [0.2, 1.1] | 3.2 | [2.1, 4.3] |
| PHQ-2 | 10.5 | [8.8, 12.2] | 1.6 | [0.9, 2.3] | 0.1 | [0.0, 0.2] | 0.0 | [0.0, 0.0] |
| GAD-2 | 11.0 | [9.3, 12.6] | 5.2 | [3.9, 6.5] | 0.1 | [0.0, 0.2] | 0.0 | [0.0, 0.0] |
|  |  |  |  |  |  |  |  |  |
|  |  |  |  |  |  |  |  |  |
| **Total** |  |  |  |  |  |  |  |  |
|  | **September** | | **October** | | **November** | | **December** | |
| Enough knowledge | 85.8 | [84.6, 86.9] | 85.8 | [84.6, 87.1] | 88.6 | [87.5, 89.7] | 92.1 | [90.9, 93.2] |
| Any visitors | 6.7 | [5.9, 7.5] | 8.9 | [7.9, 9.9] | 10.2 | [9.2, 11.2] | 6.7 | [5.7, 7.8] |
| Left home | 75.2 | [73.7, 76.6] | 72.8 | [71.2, 74.3] | 75.4 | [73.9, 76.9] | 84.0 | [82.5, 85.6] |
| Avoid crowds | 79.2 | [77.8, 80.5] | 71.3 | [69.7, 72.9] | 67.3 | [65.7, 68.9] | 62.5 | [60.4, 64.5] |
| Avoid transport | 57.3 | [55.7, 59.0] | 59.9 | [58.1, 61.6] | 62.6 | [60.9, 64.2] | 62.1 | [60.0, 64.2] |
| Use facemask | 98.4 | [98.0, 98.8] | 99.4 | [99.1, 99.6] | 99.0 | [98.6, 99.3] | 98.9 | [98.5, 99.4] |
| Unable access medication | 48.1 | [46.4, 49.8] | 53.5 | [51.8, 55.3] | 54.3 | [52.6, 56.0] | 57.2 | [55.1, 59.3] |
| Unmet healthcare need | 6.3 | [5.5, 7.1] | 4.5 | [3.8, 5.3] | 8.3 | [7.4, 9.2] | 7.9 | [6.8, 9.0] |
| Lost earnings | 8.7 | [7.8, 9.6] | 5.6 | [4.8, 6.4] | 5.1 | [4.4, 5.9] | 7.2 | [6.1, 8.3] |
| PHQ-2 | 6.8 | [5.9, 7.6] | 3.0 | [2.4, 3.6] | 4.0 | [3.4, 4.7] | 4.5 | [3.6, 5.4] |
| GAD-2 | 5.6 | [4.8, 6.4] | 2.3 | [1.8, 2.8] | 2.9 | [2.3, 3.4] | 5.1 | [4.2, 6.0] |
